# Shorter Telomere Length - A Potential Susceptibility Factor for HIV-Associated Neurocognitive Impairments in South African Woman

**DOI:** 10.1371/journal.pone.0058351

**Published:** 2013-03-05

**Authors:** Stefanie Malan-Müller, Sîan Megan Joanna Hemmings, Georgina Spies, Martin Kidd, Christine Fennema-Notestine, Soraya Seedat

**Affiliations:** 1 Department of Psychiatry, Faculty of Medicine and Health Sciences, Stellenbosch University, Cape Town, South Africa; 2 Medical Research Council of South Africa Centre for Molecular and Cellular Biology, Division of Molecular Biology and Human Genetics, Faculty of Medicine and Health Sciences, Stellenbosch University, Cape Town, South Africa; 3 Centre for Statistical Consultation, Department of Statistics and Actuarial Sciences, University of Stellenbosch, Stellenbosch, South Africa; 4 Departments of Psychiatry and Radiology, School of Medicine, University of California San Diego, La Jolla, California, United States of America; University of Cape Town, South Africa

## Abstract

The neuropathogenesis of the human immunodeficiency virus (HIV) may manifest as various neurocognitive impairments (NCI). HIV-positive individuals also have significantly shorter telomere length (TL) in peripheral blood mononuclear cells (PBMCs) and CD8+ T cells compared to HIV-negative individuals. Additionally, reduced TL has been found to be associated with chronic psychological stress. This study focused on the effects of HIV-infection and chronic stress associated with childhood trauma on telomere length, and investigated whether leukocyte TL (LTL), in particular, represents a risk factor for NCI. Eighty-three HIV-positive and 45 HIV-negative women were assessed for childhood trauma and were subjected to detailed neurocognitive testing. Blood from each participant was used to extract Deoxyribonucleic acid (DNA). Relative LTL were determined by performing real time quantitative PCR reactions as described by Cawthon et al. (2002). As expected, relative LTL in the HIV-positive individuals was significantly shorter than that of HIV-negative individuals (F = 51.56, p = <0.01). Notably, a significant positive correlation was evident between relative LTL and learning performance in the HIV-positive group. In addition, a significant negative correlation was observed between relative LTL and verbal fluency, but this association was only evident in HIV-positive individuals who had experienced trauma. Our results suggest that reduced LTL is associated with worse learning performance in HIV-positive individuals, indicating that TL could act as a susceptibility factor in increasing neurocognitive decline in HIV-infected individuals.

## Introduction

HIV/AIDS is a major health concern, particularly in South Africa, where 10.6% of the South African population is estimated to be infected with the human immunodeficiency virus (HIV) (2011 statistics) [1]. Women bear the brunt of this disease, with HIV prevalence among South African females highest in the 25–29 year age group at 32.7% [2]. Moreover, an equally devastating but unseen epidemic of abuse against women and children exists in developing parts of the world, such as South Africa, where high rates of gender-based violence, including childhood trauma, intimate partner violence and rape, predominate [3]. Research has shown that adults who experienced an increased number of traumatic events during childhood, were more prone to various health impairments, including cancer, chronic pulmonary disease, coronary artery, alcoholism, depression, and drug abuse as well as mental health problems, and cardiovascular risk factors such as obesity, physical inactivity, and smoking [4].

HIV-1, the most common and pathogenic strain of the HI-virus, invades the central nervous system (CNS) by crossing the brain blood barrier (BBB) early in the course of infection, where it can cause synaptodendritic injury through direct (e.g. viral proteins) or indirect (e.g. neuro-inflammatory) mechanisms [3]. Although the post-CART (combination antiretroviral therapy) era has seen a decrease in the prevalence of classic HIV-associated brain pathology (microglial nodules in HIV encephalitis), adverse effects of the virus on the CNS continue to have a pervasive daily impact on patients’ lives. CNS damage can be categorised according to primary pathologies, damage caused due to the presence of the virus, and secondary pathologies, consequences of immunosuppression [5]. This on-going injury may be driven by chronic inflammation, abnormal immune activation in response to HIV infection or low-level viral replication in the CNS [6] and result in persistent HIV-associated neurocognitive disorders (HAND) [6].

HIV-related damage to the CNS may manifest as neurocognitive disturbances, affecting abilities such as attention, memory, executive function, language and perceptual skills [7]. Collectively termed HAND, these are a globally occurring phenomenon across all viral clades [8]. HAND encompasses a spectrum of neurocognitive disorders, ranging from less severe disorders, such as HIV-associated asymptomatic neurocognitive impairment (ANI) and HIV-1-associated mild neurocognitive disorder (MND) to the more severe HIV-1-associated dementia (HAD) [9]. HAND has been recorded at relatively high rates in South Africa - in a recently study, 23.5% of HIV-infected South Africans were found to suffer from HAND [10]. Although the most severe form of HAND, HIV associated dementia, is less common in the CART era, high rates of mild NCI persist through stages of infection despite improved viral suppression and immune reconstitution with CART. In view of partially irreversible CNS changes that result from severe immunosuppression early in the course of illness, timely and sustained CART can be protective against adverse NCI [11].

Telomeres are nucleoprotein structures located at the ends of chromosomes, protecting them from degradation [12]. Telomeres are characterised by a variable number of tandemly repeated 5′-*TTAGGG*-3′ segments [12]. Owing to the inability of DNA polymerase to replicate the ends of linear chromosomes completely [13], telomeres shorten by approximately 20–200 base pairs during each mitotic cell division [14]. The telomerase enzyme, which adds telomeric DNA to the chromosomes, prevents excessive telomere shortening [15]. TL thus serves as a marker of cellular and biological aging [12] as well as a general index of human organismic aging [16].

Additional factors that affect the rate of telomere attrition include obesity [17], physical exercise [18], smoking [19], anti-inflammatory medications [15] and oxidative stress [13], [20]. The maintenance of TL might be the missing link translating genetic and environmental effects to aging and age related diseases. Consequently, TL shortening has been associated with an increased risk for age-related diseases and metabolic decline [21], [22], [23]. Stress hormones, inflammation and oxidative stress have been proposed as biochemical pathways that could mediate the relationship between environmental factors and TL. Furthermore, depression and chronic stress have been associated with high levels of 8- hydroxy-deoxyguanosine (8-OHdG) and a reduction in anti-oxidant enzymes [24]–[28]. Telomeric DNA is particularly sensitive to oxidative damage and in vitro activity of telomerase is inhibited by oxidative stress [29], [30]. It has been hypothesised that the continuous immune activation caused by HIV infection results in immune system exhaustion and subsequent shortening of telomeres [31]. Indeed, HIV-positive individuals have been shown to have significantly shorter telomere length in CD8^+^ T cells compared to HIV-negative individuals [32].

Recent evidence suggests that psychological and physiological stress is significantly associated with lower telomerase activity, and subsequently shorter TL [26]. Of relevance to the current study, Epel et al., 2004 [33], Tyrka et al. (2010) [34], Kananen et al., (2010) [35], O’Donovan et al., (2011) [36] and Glass et al., (2010) [37] observed associations between TL (as measured in adulthood) and childhood trauma, thereby providing preliminary evidence that early trauma accelerates cellular ageing. In addition, there is now evidence that lifetime trauma may play a role in increased cognitive impairments in HIV-positive patients [10], which may, in turn, be associated with higher mortality rates. To investigate the impact of childhood adversities on cognitive markers in adults it has to be considered that lifetime trauma is a potential co-variate that has to be considered. The mechanism by which chronic stress impacts on neurocognitive impairment (associated with HIV) is largely unknown. Identifying factors that may interact to increase neurocognitive impairment could facilitate improvements in the cognitive functioning of HIV infected individuals. Given the potential effects of chronic stress on TL, particularly during childhood, it is important to assess whether TL represents a risk factor for increased neurocognitive impairment in individuals exposed to early life trauma. We hypothesized that shorter TL would be associated with an increased susceptibility to HIV associated neurocognitive impairment in the context of early life adversity. We also investigated whether childhood trauma would have negative effects on relative LTL as described in previous studies [33], [34].

## Materials and Methods

### Ethics Statement

Written informed consent was obtained from all study participants and ethical approval was granted by the human health research ethics committee of Stellenbosch University, South Africa (ethics reference number: N07/07/153). All clinical investigations have been conducted according to the principles expressed in the Declaration of Helsinki.

### Sample

The study sample consisted of 128 women, 83 (64.84%) of whom were HIV-positive (Table 1). Socio-demographic information of all participants is provided in Table 2. Participants underwent neurobehavioural testing for neurocognitive impairment, using the International Neuropsychological Test Battery developed by the HIV Neurobehavioral Research Centre, San Diego (Table 3). A history of childhood trauma was assessed by a trained research psychologist using a retrospective self-report instrument, namely the Childhood Trauma Questionnaire - Short Form (CTQ-SF) [38]. All the tests were assessed by a trained research psychologist.

**Table 1:** Group characteristics for HIV status, childhood trauma status, mean relative LTL and LTL standard deviation (SD) values for all participants.

Effect	Level of factor	Level of factor	N	%	LTL Mean (SD)^a^
**Total**			128		0.74 (0.32)
**HIV_Status**	HIV-negative		45	35.16	0.98 (0.36)
**HIV_Status**	HIV-positive		83	64.84	0.61 (0.2)
**Childhood trauma**	No		62	48.44	0.78 (0.36)
**Childhood trauma**	Yes		66	51.56	0.7 (0.28)
HIV_Status*childhood trauma	HIV-negative	No	27	21.09	1.02 (0.38)
HIV_Status*childhood trauma	HIV-negative	Yes	18	14.06	0.92 (0.35)
HIV_Status*childhood trauma	HIV-positive	No	35	27.34	0.59 (0.19)
HIV_Status*childhood trauma	HIV-positive	Yes	48	37.5	0.62 (0.2)

a All LTL values are that of relative LTL and not absolute LTL. HIV (Human immunodeficiency virus), LTL (leukocyte telomere length), N (samples size), SD (standard deviation).

**Table 2:** Socio-demographic information of all participants.

	Frequency
**Handedness**	
Left	9 (7%)
Right	119 (92%)
**Participant age**	
Mean	29.8
Mean of HIV-positive participants	31.6
Mean of HIV-negative participants	26.33
Minimum	18
Maximum	50
**Ethnicity**	
Black	123 (95%)
Coloured	5 (4%)
**Marital status**	
Single	91 (70%)
Married	26 (20%)
Living with a partner	3 (2%)
Separated	4 (3%)
Divorced	3 (2%)
Widowed	1 (1%)
**Home language**	
English	4 (3%)
Afrikaans	16 (5%)
Xhosa	113 (88%)
Other	5 (4%)
**Annual household income**	
Less than R10 000	101 (78%)
R10 000 - R20 000	21 (16%)
R20 000 - R40 000	1 (1%)
R40 000 - R60 000	2 (2%)
R60 000 - R100 000	1 (1%)
More than R100 000	2 (2%)
**Highest level of education**	
Grade 7	1 (1%)
Grade 8	7 (5%)
Grade 9	12 (9%)
Grade 10	24 (19%)
Grade 11	49 (38%)
Grade 12	31 (24%)
**Employment status**	
Employed	46 (36%)
Unemployed	82 (64%)
**ARV treatment**	
Yes	13 (16%)
No	70 (84%)
	**Frequency**
**Handedness**	
Left	9 (7%)
Right	119 (92%)
**Participant age**	
Mean	29.8
Mean of HIV-positive participants	31.6
Mean of HIV-negative participants	26.33
Minimum	18
Maximum	50
**Ethnicity**	
Black	123 (95%)
Coloured	5 (4%)
**Marital status**	
Single	91 (70%)
Married	26 (20%)
Living with a partner	3 (2%)
Separated	4 (3%)
Divorced	3 (2%)
Widowed	1 (1%)
**Home language**	
English	4 (3%)
Afrikaans	16 (5%)
Xhosa	113 (88%)
Other	5 (4%)
**Annual household income**	
Less than R10 000	101 (78%)
R10 000–R20 000	21 (16%)
R20 000–R40 000	1 (1%)
R40 000–R60 000	2 (2%)
R60 000–R100 000	1 (1%)
More than R100 000	2 (2%)
**Highest level of education**	
Grade 7	1 (1%)
Grade 8	7 (5%)
Grade 9	12 (9%)
Grade 10	24 (19%)
Grade 11	49 (38%)
Grade 12	31 (24%)
**Employment status**	
Employed	46 (36%)
Unemployed	82 (64%)
**ARV treatment**	
Yes	13 (16%)
No	70 (84%)

**Table 3:** Ability domains that were used to measure cognition in the International Neuropsychological Test Battery.

Neuropsychological domain	Neuropsychological test
*Speed of Information Processing*	
	WAIS-III Digit Symbol
	WAIS-III Symbol Search
	Trail Making Test Part A
*Attention/Working Memory*	
	Paced Auditory Serial Addition Test
	WMS-III Spatial Span
*Abstraction/Executive Functioning*	
	Wisconsin Card Sorting Test - computer version
	Color Trails 1 and 2
	Stroop Color Word Test
	Halstead Category Test – computer version
*Learning and Delayed Recall (2 domains)*	
	Hopkins Verbal Learning Test, Revised
	Brief Visuospatial Memory Test, Revised
*Language*	
	Controlled Oral Word Association Test (FAS)
	Category Fluency (Animals, Action)
*Motor*	
	Grooved Pegboard Test (both hands)
*Screening for effort*	
	Hiscock Digit Memory Test

The CTQ-SF is a 28-item self-report inventory that provides valid screening for histories of abuse and neglect. It assesses five types of maltreatment including, emotional, physical, and sexual abuse, and emotional and physical neglect. For the present study, childhood trauma included emotional, physical, and sexual abuse and emotional and physical neglect were assessed. Bernstein and colleagues have categorised CTQ scores by type and severity but essentially, they also do stratify according to the presence of childhood trauma or it’s absence. A score of 25–31 is catergorised as no abuse or minimal abuse. A score of 41–51 is regarded as low to moderate abuse, scores from 56–68 is moderate to severe trauma and 73–125 is categorised as severe to extreme abuse [38]. Due to the small samples size in this study, we did not stratify accoding to the severity of the trauma, however we did use Bernstein’s categories to disciminate between participants with no childhood trauma and those who experienced childhood trauma. Bernstein did not describe the categorisation for scores that were between 31 and 41; we grouped participants with these scores into the absence of childhood trauma category since they are at the lower range of the spectrum. Participants with CTQ-SF scores of 41–125 were regarded as individuals that have experienced childhood trauma and those with CTQ-SF scores of 25–40 were regrded not to have experienced childhood trauma.

### DNA Procedures

DNA was extracted from whole blood via standard procedures, diluted to 25 ng/µl and amplified with QuantiFast TM SYBR Green PCR kit (Qiagen, Hilden, Germany) as described by Cawthon et al. (2002) [34], with minor modifications (different PCR kits, *HBG1* was used instead of *36B4*, different annealing temperatures). Primers specific for telomeric repeats (T) [39] and a stably expressed single copy reference gene (S), human β-globin (*HBG1*, 5′ *GCTTCTGACACAACTGTGTTCACTAGC* 3′; and HBG2, 5′ *CACCAACTTCATCCACGTTCACC* 3′) were used to amplify telomeric repeats and human β -globin, respectively. For the telomere assay each reaction included 5 µl QuantiFast 2× SYBR Green PCR Master Mix (Qiagen), 270 nM forward primer, 900 nM reverse primer, 25 ng genomic DNA and water to yield a 10 µl end volume. The composition of the human β-globin reaction was identical to the telomere assay, except that 400 nM of each forward and reverse primer was used for the β-globin assay. The reactions for the telomeric repeats and the human β-globin gene were amplified on separate 384-well plates. Each participant’s DNA sample was amplified in triplicate with both of the aforementioned primer sets for quality control purposes. If the Ct values of the triplicates of particular samples differed by more than 0.5, those samples were excluded. From the triplicate Ct values, the means were calculated for each sample and used in subsequent calculations. Amplification was performed on the ABI 7900HT platform (Applied Biosystems, Foster City, CA, USA) using the following thermal cycling profile for the telomere assay: 95°C for 5 min, followed by 40 cycles of 95°C for 10 sec and 58°C for 30 sec and a melt cycle of 95°C for 15 sec, 64°C for 15 sec and 95°C for 15 sec. The thermal cycling profile for the human β-globin gene was as follows: 95°C for 5 min, followed by 40 cycles of 95°C for 10 sec and 56°C for 30 sec and a melt cycle of 95°C for 15 sec, 64°C for 15 sec and 95°C for 15 sec.

A calibrator sample was prepared by pooling equal amounts of DNA from 40 HIV positive and 40 HIV negative participants for the construction of a standard curve. The calibrator DNA sample was serially diluted 1.68 fold per dilution, to produce a nine-point standard curve, with DNA amounts ranging from 50 ng to 0.79 ng. After amplification of the serial dilutions, a linear plot of the threshold cycle (Ct) versus the log value of the input amount of DNA (standard curve) was constructed using ABI’s SDS v.2.3 software. The efficiency of a reaction was also determined from the standard curve of that reaction. Threshold and baseline values were used as determined by the SDS v.2.3 software. All Ct values were corrected for the PCR efficiency and interplate calibration was also performed (GenEx) [40].

A validated quantitative polymerase chain reaction (qPCR) method [39] was used to determine relative telomere lengths in all samples. First, the mean telomere repeat copy number (tel, T) was normalised to a reference gene (single copy gene) (scg, S) copy number to control for differences in DNA quantity. The T/S ratio is proportional to the average telomere length. Thereafter, the factor by which the T/S ratio differed between the experimental sample and the calibrator sample was determined to provide an indication of relative average telomere length:
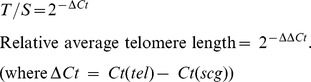



A T/S>1 indicates that the average telomere length in the sample is greater than that of the reference sample, and a T/S<1 indicates that the average telomere length in the experimental sample is less than that in the reference sample. This reference sample was a DNA sample created by pooling all the samples, at a concentration of 29.76 ng. This measure of TL is only relative and does not have a specific unit.

All participants underwent neurocognitive testing using the International Neuropsychological Test Battery developed by the HIV Neurobehavioral Research Centre (HNRC). The battery comprises tests designed to measure cognition in a variety of ability domains, namely: motor, verbal fluency, attention/working memory, speed, learning, recall, and executive functioning (testing done by a trained research psychologist). A list of tests is included in Table 3.

### Statistical Analyses

Spearman correlations were performed to determine whether an association existed between relative LTL and any of the neuropsychological test scores among the HIV-positive women (low scores are indicative of neurocognitive decline) to determine if LTL had an effect on HIV associated neurocognitive decline. Neuropsychological scores were scaled into ability domains, namely: motor, verbal fluency, attention/working memory, speed, learning, recall, and executive functioning (Table 3). A global score was also computed based on the aforementioned domains. Owing to the effects of age and education on the neuropsychological performance, age and education were added as covariates in the analyses on neuropsychological data.

Associations between possible confounding factors (age, education, body mass index [BMI], trauma sub-type, traumatic life experiences, post-traumatic stress disorder (PTSD) symptomatology and alcohol abuse) and LTL were tested using Spearman correlation tests. In addition, Spearman correlation tests were also conducted to determine if there were any associations between CD4 cell counts and LTL, viral load and LTL, ARV treatment and LTL as well as ARV treatment and neuropsychological test scores. Given that the majority of the participants (95.3%) were black, association between LTL and ethnicity was not investigated. Since 77% of the participants were infected with the clade C virus, viral clade was not corrected for as potential confounding factor in the analyses.

A two way analysis of variance (ANOVA) was conducted to determine if either HIV infection or childhood trauma, or their interaction, had a significant effect on LTL. Childhood trauma scores ranged from mild to severe, as determined by the self-reported CTQ-SF scores [38]. In view of the relatively small sample size, participants were stratified according to the presence of childhood trauma (CTQ-SF scores of 41–125) or its absence (CTQ-SF scores of 25–40) [41] and not by trauma severity. In order to determine whether LTL or childhood trauma, or the combination, had an effect on neurocognitive decline, an analysis of covariance (ANCOVA), using LTL as a covariate, was conducted.

A homogeneity of slopes ANOVA was performed to determine whether LTL and neuropsychological scores were influenced by the presence of childhood trauma. A *p*-value of ≤0.05 was considered significant for all statistical tests.

## Results

Of the 128 participants in this study, 64.84% were HIV-positive and 51.56% reported a history of childhood trauma (according to CTQ-SF cut-off scores of 41–125) (Table 1). The mean CTQ-SF score for HIV-positive individuals were 47.27 (minimum = 25, maximum = 89) and the mean CTQ-SF score for HIV-negative individuals were 42.82 (minimum = 25, maximum = 81). The majority of participants were black (95.3%), Xhosa speaking (87.6%) and unemployed (63.6%), had a low household income (less than R10 000) and had not attained Grade 12 level of education (24% had attained Grade 12) (Table 2). We tested several possible confounding factors (age, education, BMI, trauma sub-type, traumatic life experiences, post-traumatic stress disorder (PTSD) symptomatology and alcohol abuse) to establish if they had an effect on relative LTL. As expected, relative LTL was negatively correlated with age (r = −0.26, p = 0.00) and all subsequent analyses were corrected for age. None of the other variables (education, body mass index [BMI], trauma sub-type, traumatic life experiences, post-traumatic stress disorder (PTSD) symptomatology, alcohol abuse, CD4 cell counts, viral load and ARV treatment) had an effect on relative LTL. In addition, results showed that ARV treatment did not have a significant effect on neuropsychological tests scores.

**Figure 1:**
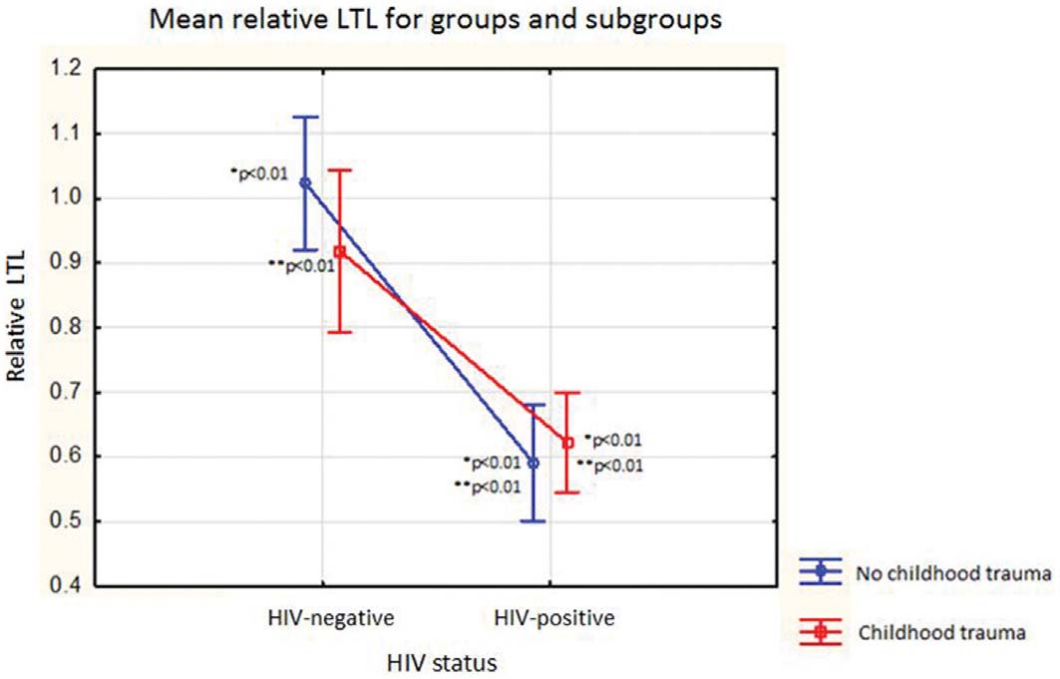
Graph displaying the group characteristics for HIV status, childhood trauma status and mean relative LTL for the groups and subgroups within the sample. No CT (childhood trauma) – participant didn’t experience childhood trauma, CT – participant experienced childhood trauma. *p<0.01 indicates significant differences between the no childhood trauma HIV-negative and no childhood trauma HIV-positive groups and no childhood trauma HIV-negative and childhood trauma HIV-positive groups. **p<0.01 indicates significant differences between childhood trauma HIV-negative and childhood trauma HIV-positive groups and childhood trauma HIV-negative and no childhood trauma HIV-positive.

**Figure 2:**
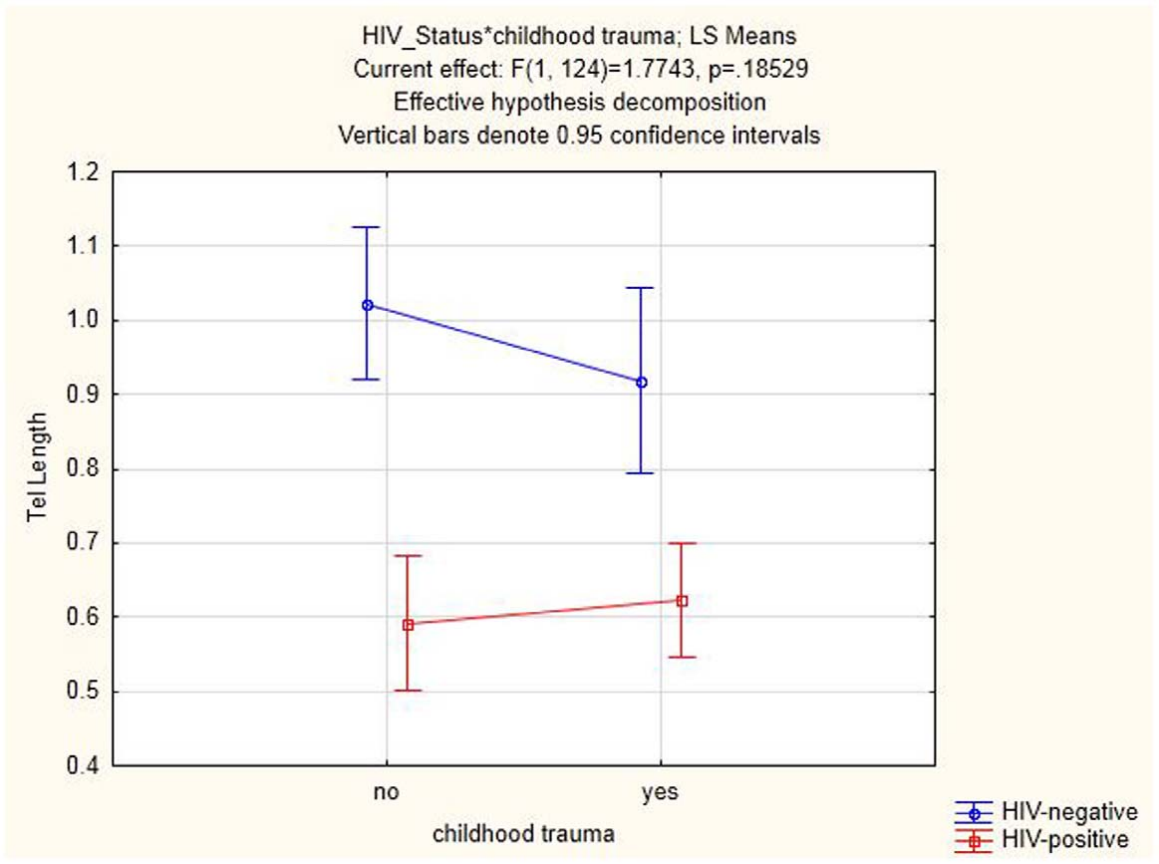
Graph depicting relative LTL in the HIV-positive group (with and without childhood trauma) as well as the HIV-negative group (with and without childhood trauma).

Figure 1 shows the mean relative LTL for all the different groups and subgroups within the sample, (statistically significant p values are included). Univariate tests of significance revealed that HIV infected individuals had significantly shorter relative LTL [LTL Mean = 0.61 (Figure 2) (Table 2)] compared to HIV negative individuals [LTL Mean = 0.98 (Figure 2) (Table 2)] (F = 51.56, p = <0.01). There was no significant independent effect of childhood trauma on relative LTL (F = 0.51, p = 0.47) (Figure 2). Additionally, there was no significant interaction effect between HIV status and childhood trauma on relative LTL (F = 1.77, p = 0.19).

In the HIV-positive group, a significant positive correlation was observed between relative LTL and learning (r = 0.26, p = 0.02); HIV-positive individuals scored significantly less in the FAS (Controlled Oral Word Association Test) learning test (*M* = 23.14, SD = 4.20) than their HIV-negative counterparts (*M* = 25.56, SD = 3.88). The correlation between relative LTL and learning remained significant in regression analysis with age included. Furthermore, a trend towards association was observed between relative LTL and recall (r = 0.2, p = 0.06). Childhood trauma did not have significant effects on either learning (F = 0.042, p = 0.84) or memory recall (F = 0.34, p = 0.56) among HIV-positive women, when relative LTL was included as a covariate (using the mean relative LTL value (0.61) of the HIV-positive group).

To determine if the relationship between relative LTL and each neuropsychological domain score was the same for the trauma/no trauma groups, a homogeneity of slopes model was applied to test whether the slopes of the two regression lines for childhood trauma status were equal (within the HIV-positive group only). There was a significant difference between the trauma/no trauma groups (no age difference between the groups) in the correlation between relative LTL and verbal fluency (F = 7.61, p = 0.007). There was no correlation in the no trauma group (r = 0.00, p = 0.98), while in the trauma group there was a significant negative correlation (r = −0.35, p = 0.01), whereby childhood trauma exposed women with shorter relative LTL performed better on tasks of verbal fluency.

## Discussion

A number of studies have investigated the immunological effects of increased telomere shortening in HIV-positive individuals. This study is the first to investigate the effects of TL on neurocognition in HIV-positive individuals, and in the context of childhood trauma. Individuals infected with HIV suffer continuous immune activation, which may lead to increased telomere shortening and subsequent accelerated immune system ageing, exhaustion of immune resources and the onset of immunodeficiency [31]. Owing to the sensitivity of telomeres to inflammation, chronic inflammation in the CNS may lead to telomere shortening in specific brain regions (white matter) as well as in PBMCs. TL could arguably be added to a list of cofactors (e.g., drugs of abuse, co-infections and age) [6] that might amplify neural injury.

Childhood abuse and other severe lifetime traumas can have lasting effects on the brain. The hippocampus is involved in learning and memory and is particularly sensitive to stress. It has been shown that adults who were abused as children have smaller hippocampal volumes [42]. In addition, a study by Gleissner and Elgar (2001) [43] found that patients with damage to hippocampal structures performed worse in verbal fluency tasks, especially tasks of semantic fluency [43].

We hypothesized that shorter relative LTL would contribute to increased cognitive impairments (associated with HIV) in the context of childhood trauma. We also investigated whether childhood trauma would have negative effects on relative LTL as described in previous studies [33], [34]. We found that relative LTL negatively influenced the learning process in HIV-positive individuals, indicating that relative LTL could possibly act as a susceptibility factor in increased neurocognitive decline in HIV infected individuals. This could be explained by the fact that excessive telomere shortening could potentially occur in white matter [44] (secondary to HIV infection) which, in turn, could lead to impairments in learning and affect memory recall ability [6]. Our results also indicated that the HIV-positive women had significantly shorter relative LTL compared to HIV-negative women; this is in line with findings from other studies that have investigated TL in PBMCs [45] and in the CD8^+^ T cell subset [32].

We did not find a significant association between relative LTL and childhood trauma. Self-reported childhood trauma ranged from mild to severe based on CTQ-SF scores [41]. Owing to small group sizes, we did not stratify childhood trauma on severity which might explain the lack of association between childhood trauma and relative LTL. In addition, telomere attrition is a relatively slow process and can best be investigated in a longitudinal study, however, this was a cross sectional study and the mean age of the participants were only 29.8 years, attributing to the lack of association between relative LTL and childhood trauma. We did find within the HIV-positive group that childhood trauma influenced the effect of relative LTL on verbal fluency (as indicated by the homogeneity of slopes model), suggesting that individuals who have experienced childhood trauma and have shorter relative LTL perform better in verbal fluency tasks. This finding is contrary to expectation; however, Harris et al. (2006) obtained similar results in a study that investigated TL, physical health, cognitive ageing, and mortality in non-demented elderly individuals [46]. They found a small significant negative association between TL and verbal fluency (based on the executive functioning test results) after they corrected for mental ability at age 11. The explanation for their finding remains to be elucidated [46].

Telomere length can be affected by a number of confounding factors, including those previously mentioned (age, education, body mass index [BMI], trauma sub-type, traumatic life experiences, post-traumatic stress disorder (PTSD) symptomatology and alcohol abuse). One of the limitations of the study is that there were variables that were not corrected for such as physical exercise [18], anti-inflammatory medications [15] and (for the small group of women on antiretroviral [ARV] treatment) the duration of ARV treatment, as this information was not available. The association between shorter telomeres and severity of NCI may reflect the fact that either or both these factors could be associated with time since diagnosis or with medication. Future studies controlling for these factors will be needed to confirm these findings. Also, information on HIV-related variables such as nadir CD4 counts, history of prior immunosuppression and time since infection was not available and could therefore not be controlled for in the analyses. The small sample size also placed a limitation on the study and prohibited sub grouping for childhood trauma severity. An additional limitation is that relative LTL was investigated in PBMCs as a whole. Studies have shown that telomere dynamics differ between subsets of PBMCs especially in different disease stages [47]–[49]. A comparative study comparing relative LTL in different brain regions (involved in the learning process) in HIV-positive and negative individuals would shed light on telomere dynamics in the brain and might explain the effect that relative LTL could have on neurocognitive decline associated with HIV infection. Further investigation of telomere length as a potential early biomarker of NCI in HIV, particularly in the setting of early life adversity, is important, both in terms of prevention and intervention efforts in neuroAIDs research. Future studies could also investigate neuroimaging endophenotypes of TL. Comparison of TL with regional brain deficits in HIV-positive individuals with NCI, those without NCI, and in HIV-negative individuals would also broaden our understanding of the specific effects of TL on HIV associated NCI and brain deficits. Moreover, larger samples would allow for stratification by childhood trauma severity and could enable detection of TL associations.

In summary, while a number of studies have investigated the immunological effects of increased telomere shortening in HIV-positive individuals, this is the first to describe an association between shorter telomeres and HIV associated NCI in the context of early life adversity. Our results indicated that LTL differed between HIV-positive and negative individuals and that there is a significant positive correlation between LTL and learning. LTL could therefore be a susceptibility factor in the development of HAND.
