# Correction: Shorter Telomere Length - A Potential Susceptibility Factor for HIV-Associated Neurocognitive Impairments in South African Women

**DOI:** 10.1371/annotation/53ec3c1c-3247-452d-85c7-576fb35bdbe3

**Published:** 2013-04-26

**Authors:** Stefanie Malan-Müller, Sîan Megan Joanna Hemmings, Georgina Spies, Martin Kidd, Christine Fennema-Notestine, Soraya Seedat

In the title, the word "Woman" should be "Women". The full, correct title and citation are:

Shorter Telomere Length - A Potential Susceptibility Factor for HIV-Associated Neurocognitive Impairments in South African Women

Malan-Müller S, Hemmings SMJ, Spies G, Kidd M, Fennema-Notestine C, et al. (2013) Shorter Telomere Length - A Potential Susceptibility Factor for HIV-Associated Neurocognitive Impairments in South African Women. PLoS ONE 8(3): e58351 

